# Determination of reliable reference genes for gene expression studies in Chinese chive (*Allium tuberosum*) based on the transcriptome profiling

**DOI:** 10.1038/s41598-021-95849-z

**Published:** 2021-08-16

**Authors:** Jing Tong, Manman Hu, Beibei Han, Yanhai Ji, Baoju Wang, Hao Liang, Mingchi Liu, Zhanhui Wu, Ning Liu

**Affiliations:** 1 Key Laboratory of Urban Agriculture (North) of Minstry of Agriculture and Rural Affairs, Beijing Vegetable Research Center, Beijing Academy of Agriculture and Forestry Sciences, Beijing, 100097 China; 2 National Engineering Research Center for Vegetables, Beijing Academy of Agriculture and Forestry Sciences, Beijing, 100097 China

**Keywords:** Biotechnology, Molecular biology, Plant sciences

## Abstract

Chinese chive (*Allium tuberosum*) is widely cultivated around the world for its unique flavor, nutrient, and medicinal values, yet its molecular mechanism on flavor formation and other metabolic pathways remains intangible. The elucidation of these complex processes begins with investigating the expression of the genes of interest, however the appropriate reference genes (RGs) for normalizing the gene expression are still unavailable in *A. tuberosum*. To fill this lacuna, transcriptome-wide screening was undertaken to identify the most stable genes according to the analysis of their FPKM values. The expression stability of the RGs was further evaluated using geNorm, NormFinder, BestKeeper, and RefFinder algorithms. The comprehensive analysis showed that *GLY1* and *SKP1*, instead of two traditionally used RGs (*eIF1α* and *ACT2*), were the most stable genes across diverse *A. tuberosum* tissues, indicating the necessity to carefully validate the stability of RGs prior to their use for normalizations. As indicated by geNorm, the normalizations with at least two RGs could give more accurate results. qRT-PCR experiments were conducted with randomly selected genes, demonstrating that normalization with a combination of *GLY1* and *SKP1* resulted in reliable normalization results. Our finding represents the first attempt toward establishing a standardized qRT-PCR analysis in this economically important vegetable.

## Introduction

Chinese chive (*Allium tuberosum* Rottler ex Spr), a perennial herb plant native to North China, has become more and more popular in the food inventory owing to its garlicky flavor, abundant nutrient, and great medicinal properties^1–3^. The first description of Chinese chive used as a vegetable plant is found in the ‘Classic of Poetry (Shijing)’, the oldest existing collection of Chinese poetry written in the Zhou dynasty (1046–256 BC) of ancient China, suggesting its long cultivation history in China^4^. In 2018, the planting area of Chinese chive reached nearly 400,000 hectares, with an estimated annual yield of 30 million tons in China^5^. Moreover, the popularity of this vegetable extends far beyond China. Japanese, Korean, Indian, and other Asian countries. For example, Chinese chive is frequently used in meat and seafood recipes in Japan.

Next-generation sequencing technology identified a number of genes linked with flavor biosynthesis as well as other metabolic processes in *A. tuberosum*^6,7^. Understanding the precise roles of these genes requires investigations on their expression profiles across different tissues and organs. However, only a few genes in *A. tuberosum* were examined by Northern blot analysis where ribosomal RNA was served as loading controls^8,9^. Unlike the many agriculturally important plants like rice, cabbage, tomato^10–12^, no report is available concerning the RG selection in *A. tuberosum*. Consequently, the absence of reliable RG(s) has dragged the application of the qRT-PCR method, which hampered the research on flavor formation as well as other metabolic processes in *A. tuberosum*.

The emergence of quantitative real-time reverse transcription-polymerase chain reaction (qRT-PCR) addresses the evident requirement for quantitative analysis in gene expressions. Compared to the Northern blotting, qRT-PCR provides a more sensitive, reproducible, and precise approach for the detection of gene expressions using a range of fluorescent report dyes that correlate the yield of PCR product with fluorescence intensity^13–15^. Gene expression data generated from qRT-PCR can be analyzed by two different approaches, absolute quantification, and relative quantification^16–18^. In absolute quantification, the expression data are determined using a standard curve generated usually based on the serially diluted standards of known concentrations^19^. However, it is labor-intensive to generate a reliable standard curve for each target gene and to include these standards in each PCR, which limits the usability of the absolute quantification in gene expression analysis^19,20^. In practice, the relative quantification method is extensively employed for the calculation of gene expressions in most laboratories. During relative quantification, the expression of target genes is calculated by the inclusion of RGs as internal controls^15,19^. Therefore, it is a prerequisite to select optimal internal control gene(s) for the normalizations under given experimental conditions.

The ideal RGs, in the relative quantifications, should be expressed constantly and stably in all examined samples regardless of experimental conditions, such as different developmental stages, biological processes, treatments, and even different organs or tissues^19,20^. However, it is a huge challenge, in reality, to identify such an RG that meets the criterion. Historically, a myriad of so-called housekeeping genes such as actins (*ACT*), glyceraldehyde-3-phosphate dehydrogenase (*GAPDH*), and ubiquitin (*UBQ*) are commonly used as internal control genes in many experiments, but recent studies have shown that their expressions are also affected by specific treatments, tissue difference, or other experimental conditions^16,17^. Besides, the veracity of qRT-PCR easily fluctuated with factors such as the quality of RNA isolation, the efficiency of cDNA biosynthesis, and PCR amplification efficiency^13^. As a result, it becomes indispensable to validate the expression stabilities and amplification efficiency of the RGs before the qRT-PCR analysis, even if housekeeping genes are employed for normalizations.

In this study, ten genes with stable expression in diverse tissues of *A. tuberosum* were screened as candidates according to the systematic survey of multiple organ-specific transcriptome analysis. Two commonly used traditional RGs, *eIF1α,* and *ACT2* in Chinese chive were also included in the comprehensive analysis by algorithms such as geNorm, NormFinder, and BestKeeper that assess the stability of genes based on the variance of quantification cycle values in each tissue samples. Computational analysis of twelve candidate genes identified that *GLY1* (DN374_c0_g1) and *SKP1* (DN253_c0_g1) displayed the most stable expression levels throughout the tissues and organs of Chinese chive. The two stable RGs were further validated by qRT-PCR, supporting that both or in combination could produce similar expression patterns as revealed by transcriptome analysis. Based on the analysis, we propose *GLY1* and *SKP1* are the preferred internal control genes for normalizing gene expression across diverse tissues of Chinese chive. To our knowledge, this study is the first analysis of the validation of RGs for the accurate determination of transcriptional patterns in Chinese chive and will facilitate expression studies on the genes associated development in this *Allium* crop.

## Results

### In silico screening of candidate reference genes from transcriptome data

In a previous study, 18 RNA-Seq libraries representing leaf, root, rhizome, mature flower, inflorescence stalk with its associated flower buds, and seed tissues of *A. tuberosum* were sequenced with Illumina Hiseq4000 platform. The raw data of all RNA-Seq samples obtained in this study were deposited in the NCBI Sequence Read Archive under the project with identification number PRJNA67394520^7^. After removal of low-quality reads, ambiguous reads, and adaptor sequences, de novo assembled transcriptome was annotated using Nr (NCBI non-redundant protein) database, which enables the identification of 223,529 tentative transcripts in *A. tuberosum*^7^.

**Figure 1:**
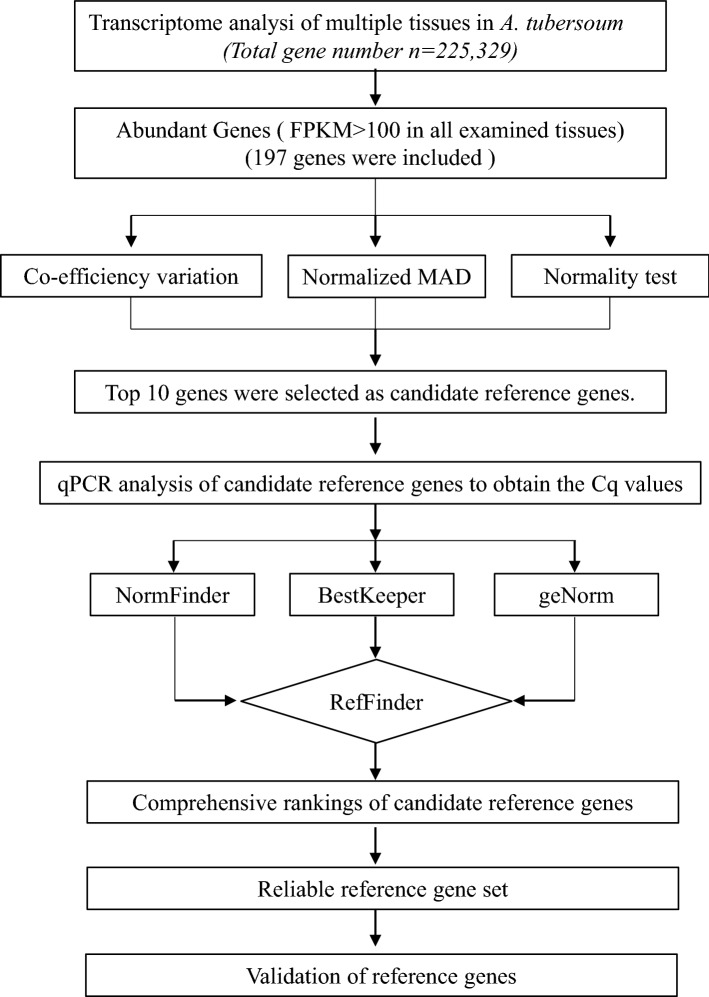
Working flow for the identification of reference genes in the experiment.

The entire workflow of the screening process is illustrated in Fig. 1. As the housekeeping genes, commonly used as RGs, are expressed at relatively high levels, the first step was attempted to identify the genes with high expressions in all *A. tuberosum* tissues. In the annotated dataset, we noted that the FPKM values of housekeeping genes such as *ACTIN, eIFs,* and *UBQ*, were high-expressing genes (average FPKM ≥ 100) in tissue samples (Supplementary Table 1). Thus, the cut-off value of median FPKM was set to 100. Accordingly, low-expressing tentative transcripts (median FPKM < 100) were eliminated, and the remaining 197 genes showed comparable expression levels as housekeeping gens (Supplementary Table 2).

**Figure 2:**
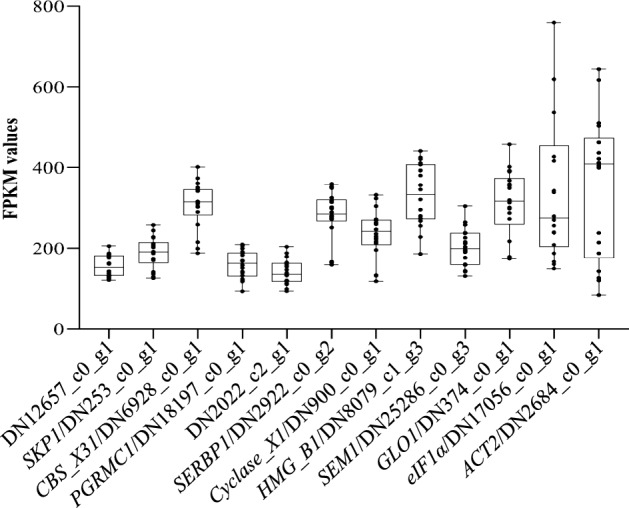
Expression levels of candidate RG and controls across various tissues based on the RNA-Seq data. The mean (horizontal line), upper and lower quartiles (white boxes), maximum and minimum values (whiskers), and each sample (black dots) are shown for individual gene.

The coefficient of variations (CV), the normalized median absolute deviations (NMADs), and the p-values measured by the *Shapiro-Wilks* were determined on the basis of the FPKM values of 197 genes. Then these genes were further ranked according to the Euclidean distance (*d*) calculated with the three parameters aforementioned (Supplementary Table 2), and primers were designed based on the sequences of the top 12 genes with the least values of Euclidean distance. DN47561_c0_g1 and DN5072_c0_g1 could not be amplified by PCR, while others were confirmed by the sequencing of their PCR products. Finally, the 10 stable genes with abundant expressions were further evaluated by using comprehensive bioinformatics analyses (Fig. 2). Additionally, based on the homologous blast search against *A. tuberosum* transcriptome, two traditional RGs (*ACT2* and *eIF1α*) which were frequently used as internal controls in Arabidopsis^21,22^, were also included in the evaluation analysis.

### Evaluation of primers’ specificity and amplification efficiency

Before experiments, gene-specific primers designed based on the assembled transcriptomes were firstly evaluated using the regular PCR method, and the resulting amplicons which contained unique bands with predicted sizes were confirmed by sequencing (Supplementary Fig. 1). Standard curves of ten candidate RGs and two controls were generated from a tenfold series dilution of at least five cDNA template concentrations and non-template control. As the R^2^ value of the standard curve represents how well the experimental data fit the regression line, the R^2^ value > 0.980 is recommended for the qRT-PCR reactions (Table 1 and Supplementary Fig. 2). Ideally, if the amount of PCR product perfectly doubled during each cycle, the theoretical amplification efficiency (E) should equal 95.4%, which indicates all templates were amplified during PCR reaction. However, in practice, an amplification efficiency of 90–105% is preferred, because qPCR was prone to be affected by suboptimal reaction conditions. As shown in Table 1 R^2^ values of other candidates and control genes, except *CBS_X3* (DN6928_c0_g1), ranged from 0.983 to 0.999, suggesting that the equations of the linear standard curves could be used for further analysis; the E values ranged from 91.57 to 109.74, which indicated the high amplification efficiency of qPCR primers and optimal reaction conditions.

**Table 1:** Details of primers and amplicons of 12 candidate reference genes.

No	Gene_id	Gene_name	NR description	Sequence (5ʹ → 3ʹ)	Amplicon size (bp)	R^2^	Amplification efficiency (%)
1	DN12657_c0_g1	N/A	N/A	CTCAACGCTCCACCGTTACT	176	0.992	105.74
				AGCCAACCAAAAATTCCACT			
2	DN253_c0_g1	*SKP1*	SKP1-like protein 1A	GGGATGCCGATTTTGTTAAA	149	0.998	92.88
				TTTCCGGATCTCTTCTGGTG			
3	DN6928_c0_g1	*CBS_X3*	CBS domain-containing protein, CBSX3	GGTGCTTTCAAATCCATGCT	152	0.969	92.20
				ATGCAAGCAATGCTATCACG			
4	DN18197_c0_g1	*PGRMC1_2*	Membrane steroid-binding protein 2-like, PGRMC1_2	TTCTAGCTCGAAGGGTCCAA	150	0.995	94.59
				GATGTCACCCAGAGCAGGAT			
5	DN2022_c2_g1	N/A	Uncharacterized protein A4U43_C06F2590	GGTAGCTGGTTCACCTTGGA	159	0.998	107.86
				AATTCCCCTGATGGTGATGA			
6	DN2292_c0_g2	*SERBP1*	RGG repeats nuclear RNA binding protein A	CAACCGAAACGAGAATGGTT	152	0.998	90.41
				CGGCATCTCCTTCAACATTT			
7	DN900_c0_g1	*Cyclase_X1*	Putative cyclase LOC109841113 isoform X1	AACAATGCAGGGCCATTAAG	167	0.999	107.10
				CCGAAATCGATGAAGAATGG			
8	DN8079_c1_g3	*HMG_B1*	HMG transcription factor B1	AAAAGCAAAAGCTGCAAAGG	150	0.983	99.13
				CACTTATCACCAGCGGCTTT			
9	DN25286_c0_g3	*SEM1*	Putative 26S proteasome complex subunit sem1-1	GGATCTTTTCGAGGATGACG	150	0.995	91.57
				TTCCTTAGCTGCAGGGAGAA			
10	DN374_c0_g1	*GLY1*	Glyoxalase I homolog 1	TCAAGGAAAGCGGTGATCTT	154	0.994	93.67
				AGGGCAATGCTTATGGACAG			
11	DN17056_c0_g1	*eIF1α*	Eukaryotic translation initiation factor 5A	TCTGACGAGGAGCATTTTGA	153	0.997	109.74
				ATGCTTGCCTGTTTTGGAAG			
12	DN2684_c0_g1	*ACT2*	ACTIN 2	GGGCATCTGAATCTCTCAGC	151	0.997	98.48
				TCGTCCGTGACATCAAAGAA			

### Expression analysis of candidate reference and control genes

**Figure 3:**
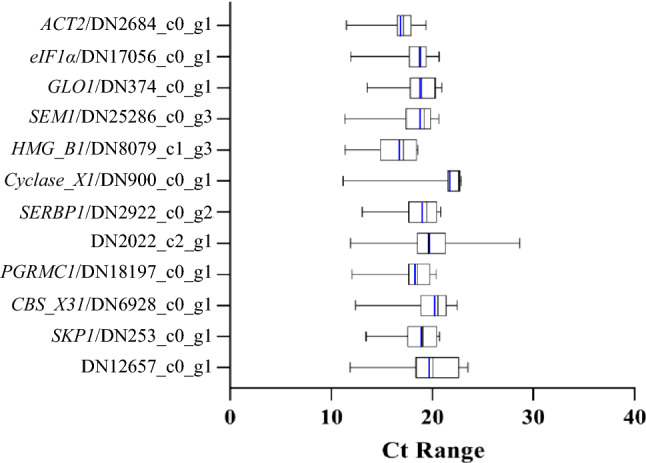
Variation of qRT-PCR Ct values for candidate RG and controls across diverse tissues in *A. tuberosum*. The vertical blue and black lines in the box show the mean and median values, respectively. The lower and upper boxes indicate the first and third quartile.

Based on the Ct values obtained from qRT-PCR, the expression levels of the selected candidate and control genes were analyzed across various tissue of *A. tuberosum*. As shown in Fig. 3, the Ct values of all examined candidate genes ranged from 11.19 to 28.6, while the average Ct value (18.51) and the median Ct value (18.96) were fairly close, suggesting that the expression data of these selected genes nearly has a symmetrical distribution. Among these candidates, it seems that *HMG_B1* was the most abundantly transcribed genes, *Cyclase_X1* was the least abundant one according to the comparison of average Ct values.

### Evaluation of expression stability of new candidate reference genes

Several software options are widely utilized to assess the variability of RGs, including GeNorm, NormFinder, BestKeeper, and RefFinder^23–26^. Thus, we employed the four programs to analyze the expression stability of candidate RGs in our qPCR experiment.

### geNorm M analysis

**Figure 4:**
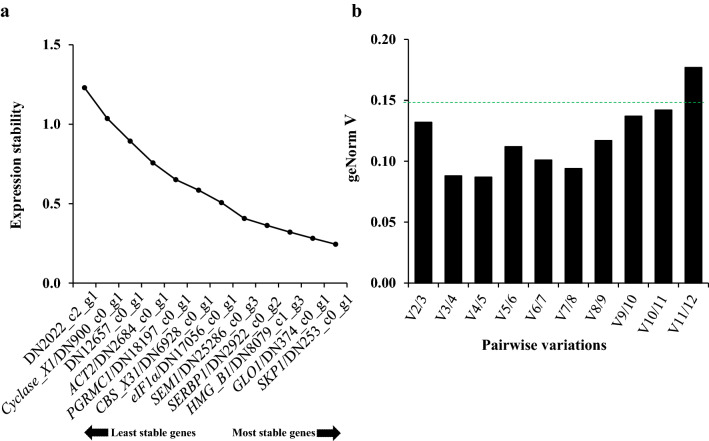
geNorm analysis of candidate RGs and controls. (**a**) Stability rankings of candidate reference and control genes based on the M values calculated by the geNorm algorithm. (**b**) Determination of the optimal number of reference genes for qRT-PCR normalization in different tissues. The variation value V was calculated by the geNorm algorithm. The default value of 0.15 (blue dash line) was taken as the threshold.

The geNorm is the most commonly used software package that helps in selecting the best RGs. The geNorm *M* value indicates the average expression stability value of remaining RGs at each step during stepwise exclusion of the least stable RG. The lower geNorm *M* values represent the more stability of the RGs, and it was recommended that the *M* value of stable RGs should below 0.5. The geNorm *M* analysis for 12 candidate genes identified that five candidates, *SKP1*, *GLY1*/DN374_c0_g1, *HMG_B1*, *SERBP1,* and *SEM1*, displayed *M* values below 0.5 (Fig. 4a), suggesting that they might be housekeeping genes across various tissues.

### NormFinder analysis

NormFinder offers a method of RGs selection that takes into account intra- and inter-group variability, while geNorm does not differentiate between groups of samples or treatments. Thus, NormFinder can prevent co-regulated genes from being selected from the candidate list. The stability value calculated by NormFinder reflects the variability of gene expression, and thus the lower value indicates more stability of RGs. Using NormFinder, *GLY1* and *SKP1 t*urned out to be the most stable genes, with expression stabilities below the cut-off value of 0.15; DN2022_c2_g1 was the most unstable gene across various tissues (Table 2). Despite the algorithm difference, the two aforementioned programs predicted that *GLY*1 and *SKP1* were the most stable genes while DN2022_c2_g1 was the least stable gene.

**Table 2:** Ranking of the candidate reference genes using NormFinder, BestKeeper, Comparative Delta Ct, and RefFinder methods.

Rank	NormFinder		BestKeeper			Comparative Delta-Ct		RefFinder	
	Genes	Stability	Genes	SD	CV	Genes	SV	Gene name	Ranking values
1	DN374_c0_g1	0.064	DN2684_c0_g1	1.43	8.47	DN2292_c0_g2	1.03	DN374_c0_g1	1.57
2	DN253_c0_g1	0.117	DN374_c0_g1	1.79	9.67	DN253_c0_g1	1.07	DN253_c0_g1	1.86
3	DN2292_c0_g2	0.183	DN253_c0_g1	1.84	9.98	DN374_c0_g1	1.07	DN2292_c0_g2	3.03
4	DN8079_c1_g3	0.364	DN18197_c0_g1	1.89	10.58	DN25286_c0_g3	1.12	DN8079_c1_g3	4.68
5	DN17056_c0_g1	0.489	DN17056_c0_g1	1.98	10.98	DN8079_c1_g3	1.15	DN2684_c0_g1	5.20
6	DN25286_c0_g3	0.499	DN2022_c2_g1	1.99	10.07	DN17056_c0_g1	1.19	DN17056_c0_g1	5.48
7	DN6928_c0_g1	0.679	DN2292_c0_g2	2.03	10.93	DN6928_c0_g1	1.22	DN25286_c0_g3	5.73
8	DN18197_c0_g1	0.690	DN8079_c1_g3	2.03	12.49	DN18197_c0_g1	1.31	DN18197_c0_g1	6.73
9	DN2684_c0_g1	1.071	DN25286_c0_g3	2.10	11.54	DN2684_c0_g1	1.57	DN6928_c0_g1	7.65
10	DN12657_c0_g1	1.296	DN6928_c0_g1	2.23	11.5	DN12657_c0_g1	1.65	DN2022_c2_g1	10.09
11	DN900_c0_g1	1.700	DN12657_c0_g1	2.69	13.82	DN900_c0_g1	1.92	DN12657_c0_g1	10.24
12	DN2022_c2_g1	3.732	DN900_c0_g1	2.70	12.98	DN2022_c2_g1	3.80	DN900_c0_g1	11.24

### BestKeeper analysis

BestKeeper assesses the stability of gene expression by comparing the standard deviation (SD) and the coefficient of variation (CV) of Ct values. Thereby, the most stable RG should have the lowest SD and CV according to the BestKeeper algorithm. The ranking result suggested that the top three stable genes were *ACT2*, *GLY1*, and *SKP1* (Table 2), indicating that they were the best RGs for normalization.

### RefFinder analysis

Depending on the input of raw Ct values, RefFinder is a web-based computational program that integrates geNorm, Normfinder, BestKeeper as well as the comparative Delta-Ct method to evaluate the RGs. Based on the rankings from four programs, it generates the geomean of the ranking values of the tested RGs. The rankings by RefFinder suggested that *GLY1*, *SKP1*, and *SERBP1* were the most stably expressed genes (Table 2), which was largely in agreement with the other three algorithms used.

### Analysis of the optimal number of reference genes for normalization

In addition to the calculation of gene stability, geNorm also calculates the pairwise variation between two sequential RGs used for normalization via geNorm V analysis. As previously proposed, the cut-off value of *V*_*n/(n*+*1)*_ is above 0.15, the inclusion of an additional RG is required. As illustrated in Fig. 4b, the value of *V*_*2/3*_ was 0.132, indicating that two references were sufficient for accurate qRT-PCR normalization. Considering the expression stabilities of candidate RGs, we thus concluded that the most stable combination was *GLY1* and *SKP1*, which could be used as reliable normalization factors for investigating the gene expression across different tissues in *A. tuberosum*.

### Validation of the reference genes

**Figure 5:**
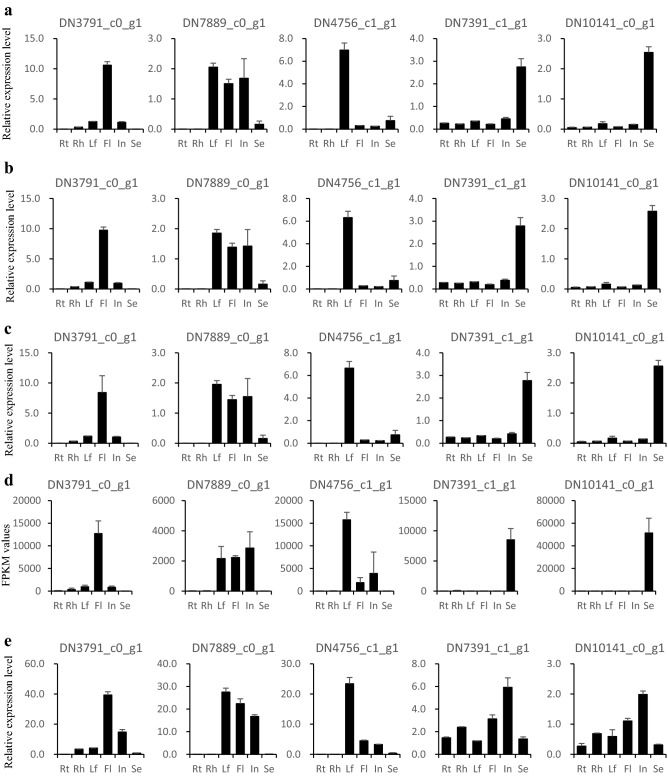
Expression analysis of five randomly selected genes across various *A. tuberosum* tissues. Expression patterns of selected genes were generated by qRT-PCR results normalized against the most stable genes, DN374_c0_g1 (**a**), DN253_c0_g1 (**b**), and in combination (**c**), or against the least stable gene DN900_c0_g1 (**e**). Expression level of selected genes in different tissues was presented according to the *A. tuberosum* transcriptome data (**d**). All these bars was represented by mean ± SD from three biological replicates.

To validate the applicability of the selected RGs, we conducted further qRT-PCR to analyze the expression profiles of several randomly selected genes from the tissue-specific transcriptome in *A. tuberosum*. Their expressions of these genes were normalized against either the most stable or unstable RGs. In general, qRT-PCR results are largely consistent with the expression patterns revealed by transcriptome analysis, when *GLY1* and *SKP1*, alone or in combination, were used as internal control genes (Fig. 5a–d). For instance, DN4756_c1_g1 shared 94% sequence similarity with the Arabidopsis *LHCB1* (Light-harvesting Chlorophyll A/B-protein 1) protein. As revealed by transcriptome data, in *A. tuberosum*, DN4756_c1_g1 was expressed in photosynthetic tissues such as the leaf, flower (sepal included), and inflorescence (with stalk). In the qRT-PCR experiments normalized against the stable RGs, we confirmed that DN4756_c1_g1 exhibited the highest expression in leaf tissues, with weak expression in flower, inflorescence, and seed tissues. Likewise, seed-abundant gene DN7391_c1_g1 encoded a putative vicilin-like seed storage protein homologous to At3g22640. Accordingly, we observed that DN7391_c1_g1 was highly expressed in seeds when using *GLY1* and *SKP1* as internal controls. Conversely, when the least stable gene, *Cyclase_X1*, was used as an internal control, DN4756_c1_g1 and DN7391_c1_g1 displayed expression patterns that differ from that of transcriptome analysis (Fig. 5e). A similar phenomenon was observed for the other three tested genes in *A. tuberosum*. Hence, the results demonstrated that, to a large extent, the RG determined the accuracy of qRT-PCR normalization. Furthermore, the results also confirmed that *GLY*1 and *SKP1* could be used as appropriate RGs in analyzing gene expression across various *A. tuberosum* tissues.

## Discussion

As one of *Allium* crops with great economic importance, Chinese chive is well-known for its unique flavor which combines garlicky and sweet odors, and for the ability to enhance the flavor of other food^2,27,28^. Several genes encoding the key enzymes in flavor production pathways have been isolated in *A. tuberosum*, but the molecular basis of flavor production remains intangible^29^. Expression profiles of several genes encoding alliinase, cysteine synthase, and serine acetyltransferase were characterized by using the Northern blot method whereby ribosomal RNA was used as loading controls^8,9^. However, studies qRT-PCR to explore expression patterns of genes in Chinese chive are still rare, and to date, no prior report on the reliable RGs has been published in *A. tuberosum* yet.

Since the relative quantification method has been widely used in the qRT-PCR analysis, reliable results are largely dependent on the selection of the appropriate RGs, expression of which is supposed to be stable in all samples, irrespective of all experimental conditions. Therefore, it is imperative to identify the stable genes that can be used as internal controls for qRT-PCR experiments. In earlier studies, the frequently used RGs in *Allium*s were usually derived from the identification of housekeeping genes that were associated with the basic cellular metabolism. For instance, *ACTIN* was used as RGs for normalizing the expression of waxy cuticle-related genes in welsh onion^30^. Similarly, an actin gene, *AsACT*, was served as internal control genes in the qRT-PCR analysis of GA-treated garlic plants^31^. However, no evidence about their constitutive expression patterns under the experimental conditions was available. In fact, the expression of the *ACT* gene could be influenced by pathogen attacks in Arabidopsis^32^. As reliable quantification of gene expression mainly depends on an accurate normalization, the selection of RGs is one of the crucial factors in investigating gene expressions under given experimental conditions.

High-throughput RNA-Seq technologies facilitate gene expression analysis especially for those species whose genomes are still unavailable. Apart from the sequence information of all the transcribed genes, the assembled transcriptome provides the landscape of gene expressions at the genome-scale, which permits the identification of the genes with stable expressions across different experimental conditions or samples. Depending on the calculation methods employed in the transcriptome analysis, gene expressions can be presented as FPKM (Fragments per kilobase per million mapped reads) value or others. Therefore, according to the FPKM values, researchers can examine the expression stability using the mean expression value, standard deviation, coefficient of variation (CV), and normalized median absolute deviation (NMAD). The CV reflects the extent of variability in relation to the mean of the expression levels, and it was successfully used to identify the best RGs from the transcriptome dataset^33–35^. However, the CV is simply calculated based on the means, which is less susceptible to the deviations caused by the outliers. Thus, the NMAD can be included to evaluate the spread of the distribution according to the medians of the expression levels, which provides a robust alternative criterion.

It was not surprising that different screening criteria might lead to contrasting results. As observed in our analysis, the rankings according to the CV values suggested that DN47561_c0_g1, DN12657_c0_g1, DN6928_c0_g1, DN253_c0_g1/*SKP1*, and DN18197_c0_g1 were the top 5 stable genes; however, DN5298_c0_g2, DN47561_c0_g1, DN6928_c0_g1, DN253_c0_g1/*SKP1*, and DN2292_c0_g2 were ranked as the top 5 stable genes when these RG candidates were scored by NMAD values. Thus, *DN47561_c0_g1, DN6928_c0_g1, DN253_c0_g1/SKP1* seemed to be the promising RG candidates because they could meet the two screening criteria. However, other candidates only met one of the criteria mentioned above, which made it difficult to pick up the ideal RG for further analysis. To solve the problem, CV and NMAD, together with the normality test of gene expressions (*p*), should be taken into account. Assuming that CV, NMAD, and *p* had equal contributions to the evaluation of RG’s expression stability, the Euclidean distance (*d*) between the three parameters could be used as an effective measure in the RG selections from the huge RNA-Seq dataset.

A Venn diagram was generated with the top 20 stable RG candidates ranked by their CV, NMAD, or *d* values (Supplementary Fig. 3). As revealed by the Venn analysis, 12 RG candidates were shared by three rankings lists, suggesting that they might be the ideal RG candidates which met all three criteria. 19 genes were shared between *d* and CV ranking lists, while only one gene was unique for the CV ranking list. Likewise, 13 genes appeared on both NMAD and *d* ranking lists, whereas 7 genes formed a unique group that was specific to the NMAD ranking list. In our analysis with top 20 RG candidates, the outcomes with the CV and *d* criteria were similar to a large extent, though the ranking orders of some RGs were different; on the contrary, the NMAD might serve as a more restrictive criterion, and nearly half of genes appeared in the overlapping between both *d* and NMAD ranking list, implying that NMAD criterion alone is not sufficient to evaluate the stability of RG expressions. As the Euclidean distance can balance the effects from three parameters, we preferred to use *d* values instead of CV or NMAD to evaluate the first-round screenings, and in the subsequent bioinformatics analyses, the selected 10 RG candidates, which were further confirmed by PCR experiments, exhibited better expression stability compared to that of two traditional RGs.

By combining some bioinformatics tools, NormFinder, geNorm, BestKeeper, and RefFinder, a series of RG candidates were identified in non-model species, such as cotton, onion, soybean, and garlic^36–39^. In addition to traditionally used RGs—*eIF1α* and *ACT2*, the top 10 potential RGs identified from transcriptome analysis were quantitatively evaluated with the programs aforementioned. Surprisingly, the majority of RG candidates were associated with either ubiquitin or ribosomal pathways except that SEM1 might encode one of the components in the 26s proteasome^40^. Ubiquitin genes were often used to normalize the quantification of gene expression due to their abundance and universal expressions in diverse samples. Among the top 200 RGs, we found at least 13 genes that were associated with ubiquitin pathways, but the ranking list suggested that they did not appear to be the best RGs as some expression variations of these genes were detected (Supplementary Table 1). It has been generally accepted that, despite housekeeping roles in the intracellular protein degradation, the ubiquitin–proteasome system was involved in selective proteolysis, and therefore the process was tightly controlled and dynamically regulated^41^. Similarly, it becomes controversial whether the 18s or 28s ribosomal genes are perfect RGs. On one hand, like ubiquitin genes, transcription of some ribosomal genes was also affected by biological factors^26^; On the other hand, the ribosomal RNAs are not polyadenylated, which makes it difficult to investigate their expression when dealing with cDNA from total RNA with oligo-dT primers. It was reported that the use of ribosomal genes in expression studies could give erroneous normalizations compared with the use of other RGs^42,43^. Consistent with previous findings, in this study, the top 10 RG candidates could thus be used to normalize expression levels of genes of interest despite that most of the candidates might be not related to ubiquitin or ribosomal pathways.

The comprehensive rankings suggested that, of the 12 genes, *GLY1*/DN374_c0_g1 was the most stable gene, followed by SKP1/DN253_c0_g1; on the contrary, Cyclase_X1 was thought to be the least stable one. Interestingly, we found that the housekeeping genes, *eIF1α* and *ACT2,* were not constantly expressed despite that their Arabidopsis homologs were widely used as internal controls in molecular biological experiments^43,44^. As revealed herein, the most stable genes identified in our analysis also included genes involved in signaling pathways. For example, DN253_c0_g1 encoded a putative S-phase kinase SKP1, which might mediate the ubiquitination of proteins involved in cell cycle progression^45^. DN8079_c1_g3 was ranked as the fourth stable gene by RefFinder, though it might encode a putative a high mobility group protein (HMG) which could regulate chromatin remodeling and ultimately gene transcription^46^. Therefore, the results reminded us that the empirically selected ‘housekeeping’ genes could be unsuitable as the internal controls, and their stabilities should be carefully evaluated before experiments.

Compared to the other algorithms, only geNorm provides a method to define the optimal number of RGs for accurate normalization of gene expression via the pairwise variation V analysis. The cut-off Vn/n + 1 value of 0.15 is recommended, indicating that the preferred primer number is n; otherwise, an additional RG should be included. In our experiments, the V2/3 was below the threshold value of 0.15, suggesting that two RGs were sufficient for data normalization. When using two RGs alone and in combination, the qRT-PCR analysis of randomly selected genes showed similar expression patterns in our experiments. However, it is still recommended to always use at least two RGs to avoid substantial errors, because we could not deny the possibility that these stable genes might participate in other biochemical pathways other than plant development.

We analyzed the expression data of five randomly selected genes in *A. tuberosum* normalized against either the most or the least stable genes according to the comprehensive rankings given by the RefFinder algorithms, demonstrating that transcriptional patterns analyzed with the stable RG were almost the same as observed in transcriptome analysis. On the contrary, when the raw data normalized with the least stable gene, the qRT-PCR analysis displayed distinct expression patterns of target genes, which could lead to biased interpretations of gene functions. Thus, our results suggested that the importance of appropriate RGs, which will enable more accurate and reliable normalizations in gene expression analysis.

In conclusion, we finally demonstrated the fidelity of *GLY1* and *SKP1* as the optimal candidate RGs in qRT-PCR experiments with diverse *A. tuberosum* tissues. This is the first report on the selection of RGs in *A. tuberosum*, which will benefit the study of gene expressions and other related subjects in Chinese chive. Nevertheless, we should be cautious when including the two RGs into qRT-PCR experiments in *A. tuberosum*, because their expression stability was only verified at the developmental aspect and could be affected by other experimental conditions.

## Materials and methods

### Plant materials

The Seeds of *A. tuberosum* cultivar ‘791’ were bought from the Jingyan Yinong (Beijing) Seed Sci-Tech Co. Ltd, and all subsequent experiments were conducted according to the guidelines and regulations of the Beijing Vegetable Research Center (BVRC), Beijing Academy of Agriculture and Forestry Sciences. The seeds of Chinese chive were germinated on a 32-cell tray with granulated rockwool, and seedlings were irrigated with ¼ strength of Hoagland’s medium once a day. After 2-month growth, seedlings were transferred to a hydroponic cultivation system in a controlled greenhouse located in the BVRC. The plants were supplied with the nutrient solution, and the composition formula as previously described^7^. Tissue samples of 3-year-old seedlings were harvested from the mature leaves, roots, rhizome, mature flowers, inflorescences, and seeds. All tissue samples were fleshly collected from at least 10 individual plants and were frozen immediately in liquid nitrogen until use. Sample collections were performed on separate days for three replicates. In the course of experiments, we complied with the IUCN Policy Statement on Research Involving Species at Risk of Extinction and the Convention on the Trade in Endangered Species of Wild Fauna and Flora. The ethical approval was deemed unnecessary according to the Decree of the State Council of China No. 204.

### RNA-Seq and transcriptome de novo assembly

The 18 RNA-Seq libraries representing the six tissues were prepared were sequenced at the Illumina NovaSeq 6000 platform to an average depth of 50 million reads per sample. Sequence reads were filtered using SeqPrep (https://github.com/jstjohn/SeqPrep) and Sickle (https://github.com/najoshi/sickle) to remove the low-quality and adaptor sequences. Clean reads were assembled via the Trinity de novo assembly program (https://github.com/trinityrnaseq/trinityrnaseq)^47^ and TransRate (http://hibberdlab.com/tran-srate/)^48^. For annotation purposes, sequences were handled with CD-HIT (http://weizhongli-lab.org/cd-hit/) program to reduce the transcript redundancy^49^, and finally, the assembly quality was evaluated using BUSCO (Benchmarking Universal Single-Copy Orthologs, http://busco.ezlab.org) program with default configurations^50^. All raw data were deposited NCBI Sequence Read Archive under the project with identification number PRJNA67394520, and the assemble transcriptome is accessible at a public server (https://doi.org/10.6084/m9.figshare.14820201).

### Screening of candidate reference genes from transcriptome data

Candidate RGs were screened according to the previously described method with modifications^37^. Briefly, based on the transcriptome data from the six tissues, four parameters were adopted to evaluate the stability of gene expressions: (1) The coefficient of variation (CV), which detects the extent of variability in relation to the mean of the expression levels; (2) The normalized median absolute deviation (NMAD). In statistics, median absolute deviation (MAD) is commonly used for assessing the spread of the distribution according to the medians and is less susceptible to the deviations by outliers. The MAD was further normalized against the median, which could reflect the variability of gene expressions based on the medians. Compared to the CV that is often affected by outliers, and the NMAD provides a robust alternative; (3) Normality test. The p-value measured by the *Shapiro–Wilks* hypothesis indicates whether the expression data does fit the normal distribution, and in the test, p-value < 0.05 means a significant departure from normality. Most importantly, FPKM values were also taken into account. Because genes with low expression abundance are unsuitable as RGs, we used FPKM ≥ 100 as the cut-off value for candidate selection.

The ideal RGs should have lower or similar statistical variations across samples, which was indicated by low CV and NMAD values. Moreover, their expression data also should pass the normality test, which allows that with 95% confidence they fit the normal distribution. To meet the criteria proposed herein, RGs were finally ranked according to the calculation of Euclidean distance (*d*), and the formula was as follows: $d=\sqrt{{CV}^{2}+{NMAD}^{2}+{(1-p)}^{2}}$. After comparisons, the top 10 genes with the least values of Euclidean distance were selected for further analysis.

### RNA extraction, the cDNA synthesis

Total RNA isolation was performed as described elsewhere^51^. Briefly, total RNA was extracted with Transzol (Transgen Biotech, China) according to the manufacturer’s instructions. Briefly, tissues of *A. tuberosum* were homogenized in liquid nitrogen and the frozen powder was lysed using Transzol extraction buffer. 1/5 volume of chloroform was added before 5-min centrifugation at 12,000*g*. The aqueous containing nucleic acid was precipitated with an equal volume of isopropanol by centrifuging at 12,000*g* for 10 min. The resultant pellets were washed in 75% ethanol and resuspended in RNase-free water. After DNase I digestion, the clean total RNAs were stored at − 80 °C freezer. RNA quantity and quality were examined using a NanoDrop 2000 spectrophotometer (Thermo Fisher Scientific, USA) to ensure structural integrity for further experiments.

First-strand cDNA synthesis was conducted by using the EasyScript First-Strand cDNA Synthesis SuperMix kit (Transgen Biotech, China) according to the user’s manual. In summary, 1 µg of total RNA was mixed with 1 µl oligo (dT)_18_ primers (10 mM), and RNase-free water. The mixture was incubated for 5 min at 65 °C before being chilled on ice for 3 min. Subsequently, 4 µl of the first-strand buffer, 1 µl of gDNA remover, and 1 µl of reverse transcriptase were added to finalize the reaction. After 1-h incubation at 42 ℃, the reaction was inactivated for 10 s at 70 °C. The cDNA diluted by fivefold with PCR-grade water was ready for use.

### Quantitative real-time PCR

The amounts of individual genes were estimated with gene-specific primers by quantitative real-time PCR analysis with a real-time PCR instrument Roche LightCycler 480 and SYBR Green mixture (Toyobo, Japan) as previously described^52^. Briefly, primer pairs showing a single amplified product with the predicted sizes were chosen for further qRT-PCR experiments. Each PCR reaction mix consisted of 10 µl of SYBR Green Supermix, 0.5 µl of forward and reverse primers (10 mM), and 2.5 µl fivefold diluted template cDNA. Finally, the resulting reaction volume was made up to 20 µl by adding 7 µl PCR-grade water. The PCR cycling was performed as follows: 5 min at 94 ℃ followed by 40 cycles of 15 s at 94 ℃, 10 s at 58 ℃, 30 s at 72 ℃, and finally, 1 round of 60 s at 60 ℃, and the melting curve cycling consisted of 15 s at 95 ℃, 1 min at 60 ℃, 30 s at 95 ℃, and 15 s at 60 ℃. For qPCR efficiency, Based on the standard curve generated with serial dilutions of the pooled cDNA template, the amplification efficiency (E) of each primer pair was calculated using the equation: E% = (10^–1/slope^ − 1) × 100. For the validation experiment, the relative expression level was calculated by the 2^−∆ct^ method with selected RGs, where ΔCt is the difference in the threshold cycles between the target and the RG. All qRT-PCR reactions were performed in technical triplicate. The specific primers used in this experiment are shown in Supplementary Table 3.

### Expression stability analysis of potential reference genes

The expression stability of the candidate RGs was evaluated by using geNorm (https://genorm.cmgg.be/), NormFinder (https://moma.dk/normfinder-software/), BestKeeper (https://www.gene-quantification.de/bestkeeper.html), and RefFinder (https://heartcure.com.au/) statistical algorithms^23–26,53^. The qBase+ software with geNorm analysis was employed^26^, while NormFinder and BestKeeper analyses were performed with free add-ins for Microsoft excel^23,24^. RefFinder integrated the above three algorithms and the Delta Ct method is a free, web-based service for comprehensive ranking of RGs^25^. Based on the sequential pair-wise comparisons, geNorm calculates the stability value M and the variation value V, by which the stability of each gene and the number of optimal RGs is determined. NormFinder ranks individual candidates according to their stability value. BestKeeper determines the optimal references by employing pair-wise correlation analysis of all pairs of candidate genes. Using the LightCycler 480 Relative Quantification Software module (Roche, USA), quantification cycle (Cq) values were obtained and converted into relative quantities by using standard curves, then applied for evaluation. The relative expression levels were imported into geNorm and NormFinder, whereas BestKeeper and RefFinder analyses were conducted with the raw non-transformed Cq values.

## Supplementary Information

Supplementary Tables.

Supplementary Figures.
