# Supplementary material for: Determination of reliable reference genes for gene expression studies in Chinese chive (Allium tuberosum) based on the transcriptome profiling
Source: Sci Rep. 2021 Aug 16;11:16558. doi: 10.1038/s41598-021-95849-z (PMC8367972; doi:10.1038/s41598-021-95849-z)
Supplement: Supplementary file 2 — Supplementary Figures. [file 41598_2021_95849_MOESM2_ESM.pptx]

## Slide 1
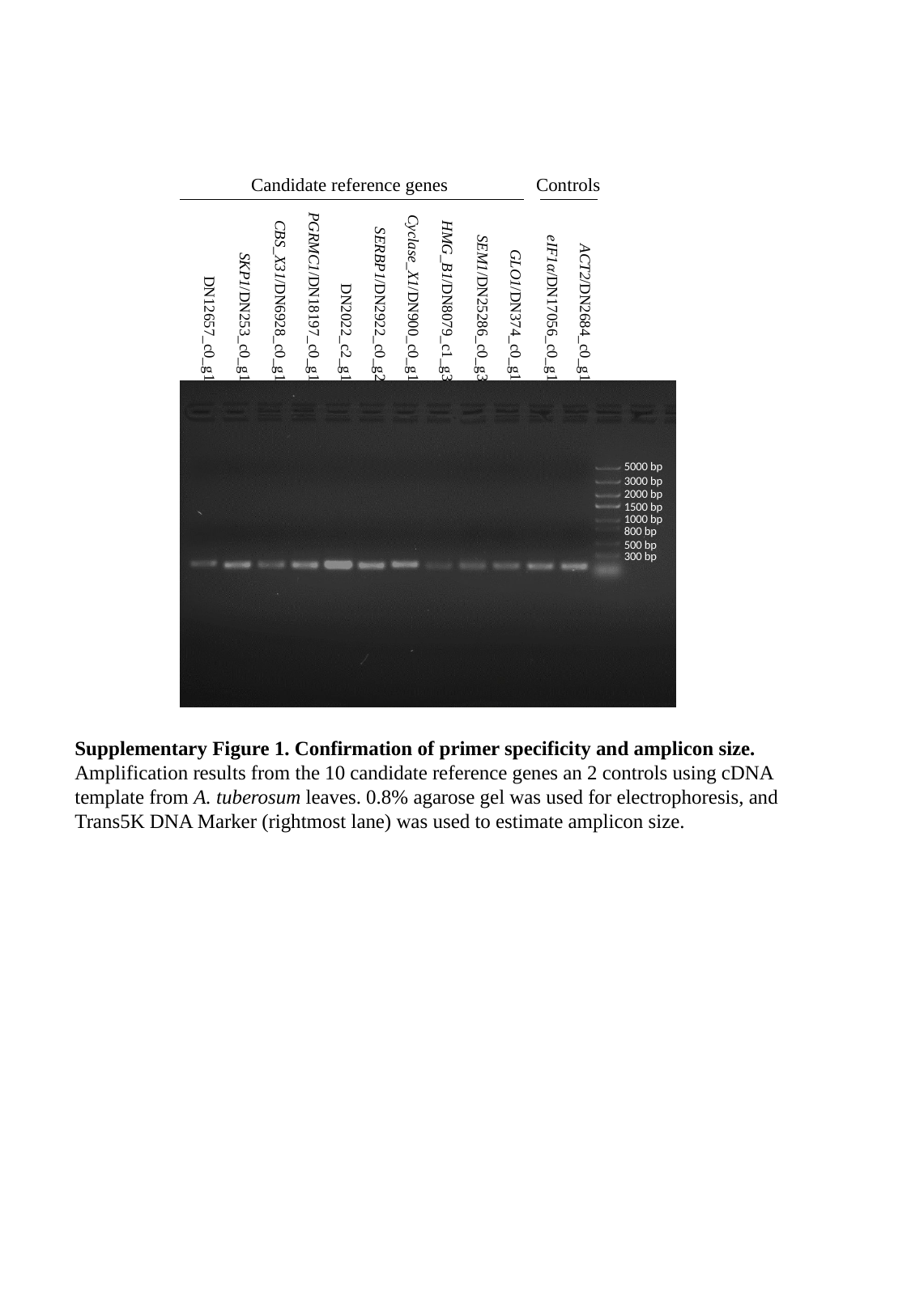

Candidate reference genes
Controls
DN12657_c0_g1
SKP1/DN253_c0_g1
CBS_X31/DN6928_c0_g1
PGRMC1/DN18197_c0_g1
DN2022_c2_g1
SERBP1/DN2922_c0_g2
Cyclase_X1/DN900_c0_g1
HMG_B1/DN8079_c1_g3
SEM1/DN25286_c0_g3
GLO1/DN374_c0_g1
eIF1α/DN17056_c0_g1
ACT2/DN2684_c0_g1
5000 bp
3000 bp
2000 bp
1500 bp
1000 bp
800 bp
500 bp
300 bp
Supplementary Figure 1. Confirmation of primer specificity and amplicon size. Amplification results from the 10 candidate reference genes an 2 controls using cDNA template from A. tuberosum leaves. 0.8% agarose gel was used for electrophoresis, and Trans5K DNA Marker (rightmost lane) was used to estimate amplicon size.

## Slide 2
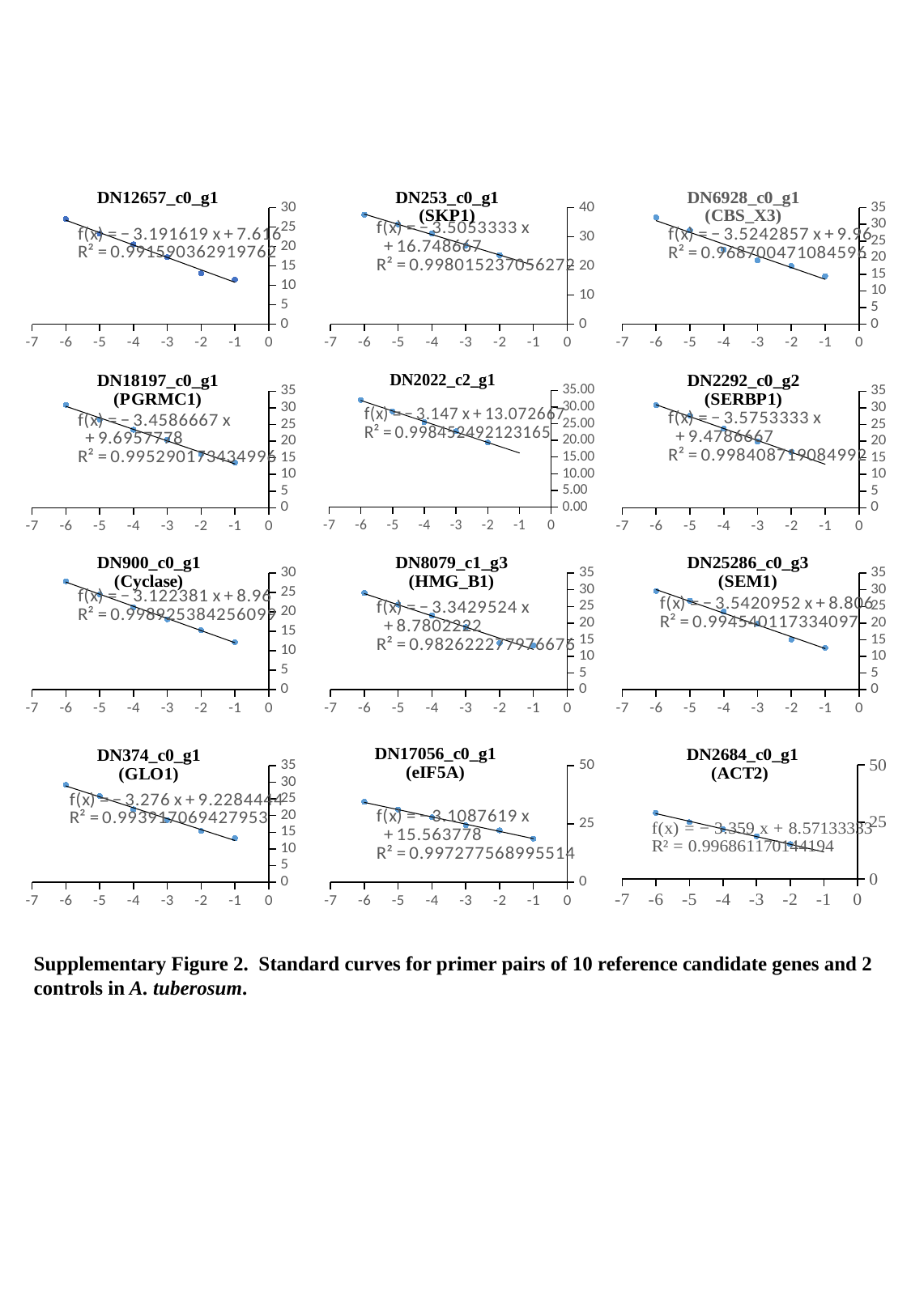

### Chart: DN12657_c0_g1
| Category | |
|---|---|
### Chart: DN253_c0_g1
(SKP1)
| Category | |
|---|---|
### Chart: DN6928_c0_g1
(CBS_X3)
| Category | |
|---|---|
### Chart: DN2022_c2_g1
| Category | |
|---|---|
### Chart: DN18197_c0_g1
(PGRMC1)
| Category | |
|---|---|
### Chart: DN2292_c0_g2
(SERBP1)
| Category | |
|---|---|
### Chart: DN900_c0_g1
(Cyclase)
| Category | |
|---|---|
### Chart: DN8079_c1_g3
(HMG_B1)
| Category | |
|---|---|
### Chart: DN25286_c0_g3
(SEM1)
| Category | |
|---|---|
### Chart: DN374_c0_g1
(GLO1)
| Category | |
|---|---|
### Chart: DN17056_c0_g1
(eIF5A)
| Category | |
|---|---|
### Chart: DN2684_c0_g1
(ACT2)
| Category | |
|---|---|Supplementary Figure 2. Standard curves for primer pairs of 10 reference candidate genes and 2 controls in A. tuberosum.

## Slide 3
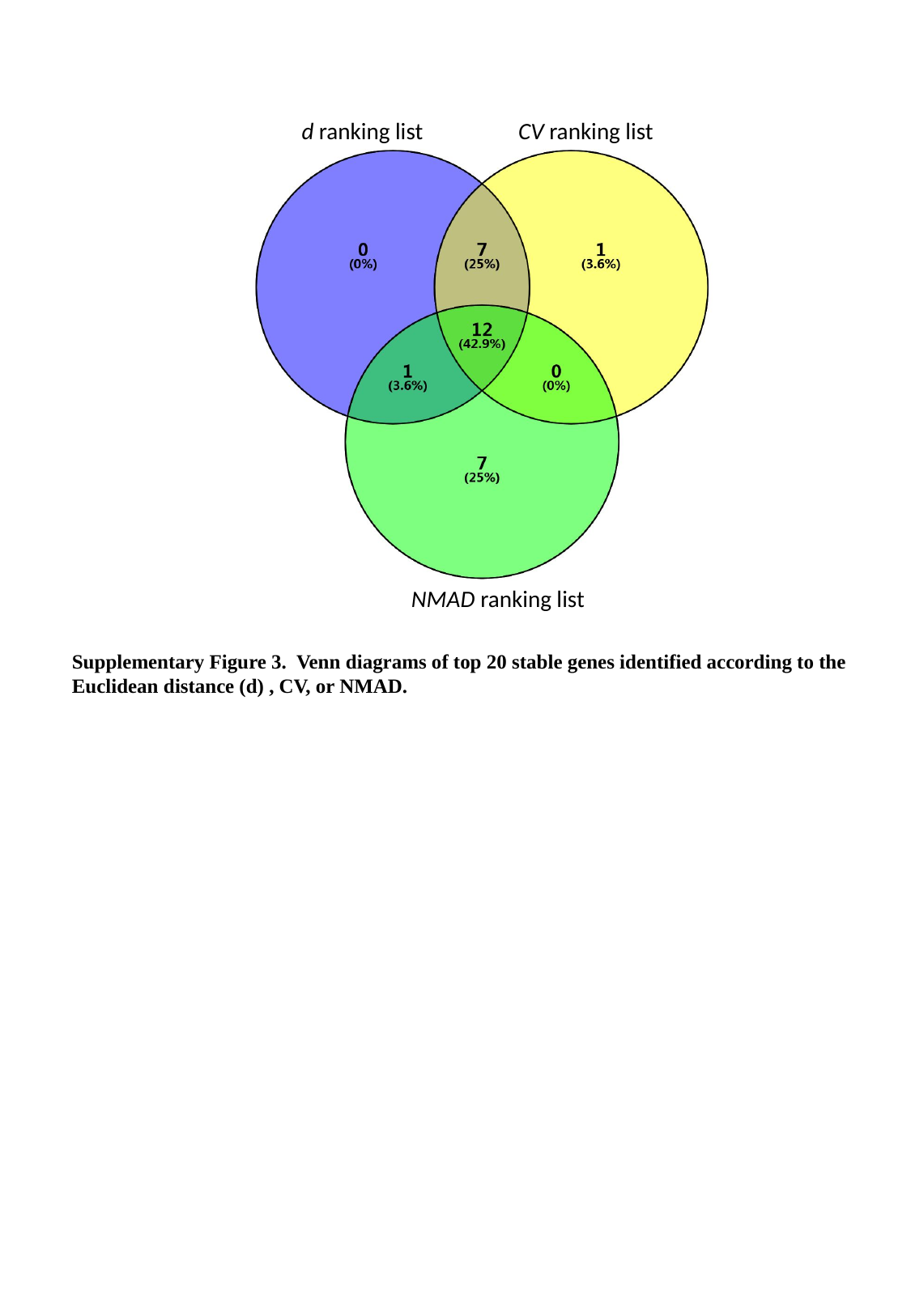

d ranking list
CV ranking list
NMAD ranking list
Supplementary Figure 3. Venn diagrams of top 20 stable genes identified according to the Euclidean distance (d) , CV, or NMAD.
